# Transformation of public administration in the sphere of forensic science activity in Ukraine under martial law

**DOI:** 10.1016/j.fsisyn.2026.100707

**Published:** 2026-06-09

**Authors:** Nataliia Martynenko

**Affiliations:** Department of Law and Public Administration, Kyiv National University of Construction and Architecture, 31, Povitroflotsky Avenue, Kyiv, 03037, Ukraine

**Keywords:** Forensic science, Public administration, Martial law, Resilient governance, Standardization, Accreditation, Digitalization

## Abstract

The current shortcomings in the public administration of forensic science activities in Ukraine have been analyzed. The work of state specialized institutions in wartime conditions has been examined. The technical and political significance of the digitalization of forensic science activities is clarified. Attention is paid to the problems of instability in the management and staffing of forensic institutions. The issue of standardization and accreditation of forensic institutions is examined. The importance of expanding international cooperation is emphasized. The proposed reform initiatives cover both the organizational and managerial aspects as well as the technological aspects of the development of the forensic science system, forming a comprehensive model for its adaptation to contemporary security, social, and technological challenges. Ensuring impartial development of norms and their equal application to all participants in the expert services market is possible only if there is a clear delineation of the functionality: formation of public policy, regulation and control, as well as management of forensic institutions. Such a division of powers minimizes the risks of unequal conditions for different subjects. Effective mechanisms for public administration of the field of forensic science expertise in conditions of martial law should be contextually relevant and capable of implementing changes without resistance.

## 1 Introduction

The problems that Ukraine and its law enforcement system are facing today are extremely large-scale and extraordinary. The modern paradigm of public administration is being transformed under the influence of severe military challenges and the strategic imperative of European integration. The complex political and economic situation in the country necessitates the search for new mechanisms of public administration in general and the sphere of forensic science activity in particular. It is important that the system of forensic science examination actively develops and evolves, despite the exceptionally difficult conditions of the harsh reality of wartime.

The traditional approach to administration of forensics in Ukraine, based on departmental principles and outdated resource provision, is in urgent need of theoretical, methodological, and institutional modernization. This is enhanced by demand for ensuring international cooperation in the field of forensic science aimed at preventing, detecting and investigating transnational crimes, including war crimes, unifying approaches to the exchange of forensic information and ensuring human rights. The urgency of the present study is justified by the inconsistency of legal, organizational, personnel, information, financial, and security mechanisms with modern requirements of public administration in the studied area. The existing organizational principles of certification of forensic scientists, as well as the opacity and insufficient funding of state specialized institutions [1]; absence of a unified scientific and methodological approach to conducting examinations [2]; departmental disunity; limited competition due to monopoly [3]; the ramifications and inconsistencies of regulatory and legal support for forensic science activities [4] create risks for the independence, objectivity, and scientific validity of forensic examination.

Ukraine's wartime experience demonstrates the need for a unified, digitally integrated, resilient forensic science governance system. Research aimed at forming a systemic administration paradigm in the field of forensic science activity, which should ensure its unity, quality, transparency, reliability, impartiality, balance, and compliance with the requirements of the strategic development of Ukraine, is becoming more opportune and relevant.

## 2 Literature review

Scholarly thought is increasingly embracing the impact of globalization on all areas of socio-political activity, including forensics. The way forward should be ambitious and not focused on organization, technical details or protocols. Most importantly, there should be a globally shared understanding of forensic science and its principles [5]. Scholars emphasize the growing attention to the Sustainable Development Goals in the field of forensics which has long since moved beyond the classical role of facilitating criminal investigations and supporting the justice system and increasingly encompasses humanitarian, peacekeeping and security activities [6].

The focus is on the systemic underestimation of forensic science activities in the field of public safety, where the priority given to police work often overshadows the role of scientific methods that ensure the accuracy of investigations, prevent miscarriages of justice, and contribute to the long-term security of society [7]. Scholars conclude that forensic science should not be viewed solely as a commercial activity, or a purely scientific field, or an academic discipline [8]. Although it intersects with all of these areas, its basic essence is that it is primarily a public service that plays a key role in ensuring fair justice and maintaining public confidence in the entire criminal justice system [8]. Therefore, the administration, financing, and strategic development of this area should correspond to its high social mission and civic responsibility [8]. In general, any management involves establishing rules that help: to verify input and output data (i.e. control mechanisms); to divide and allocate tasks (i.e. coordination mechanisms); to reconcile competing interests (i.e. incentive mechanisms): and to reduce the vulnerability of relationships (i.e. trust mechanisms) [9]. A realistic path to substantial changes in the field of forensic science is seen in the development of a unified, integrated strategy that brings together all stakeholders, various disciplines and sectors, and takes into account socio-cultural characteristics at local, regional, and global levels [10].

The formal designation of the Ministry of Justice of Ukraine as the sole body responsible for formulating public policy in the field of forensic examination without conducting a deep institutional transformation will not eliminate the existing systemic problems, and in some cases may even exacerbate them [11]. The main reason is that the Ministry of Justice of Ukraine combines several incompatible functions, acting as a developer of regulatory rules for the entire field, and the direct head of the largest network of state expert institutions [11].

The message formed by the authors encourages reflection on the need to conduct systematic scientific research in the field of public administration of forensic science activities.

## 3 Methods and material

The application of the principle of comprehensiveness made it possible to examine the main aspects and facets of public administration of forensic science activities in Ukraine taken together and to identify existing problems based on the sum of facts. The principle of specificity was implemented by establishing the facts and taking into account all material circumstances affecting the work of specialized state institutions under the complex conditions of the full-scale war, in particular: logistical barriers and challenges related to the transportation of physical evidence and materials; operations in areas subject to ongoing shelling or where there is a risk of landmines; a shortage of methodological resources, which requires targeted research, the integration of international standards into forensic science activities, and the updating of approaches to their implementation in light of current practical needs; the preservation of objects of study; countering the enemy in cyberspace; integration of modern technologies into global trends in the digital transformation of forensic science activities; forced relocation of forensic institutions and concentration of a significant number of cases in safer regions; the growing role of international cooperation, etc. This principle helped identify the specific mechanism linking general patterns to their individual manifestations, and view the object of study as a component of a broader system of which it is a part.

The method of systems analysis helped clarify the problem of instability in the leadership of state specialized institutions and the turnover of specialists. The comparative legal method was used to compare approaches to strategic vision of the development of forensic science activity.

The axiomatic approach was aimed at ensuring effective public administration of the forensic sector in a democratic law-governed state that is at war. It was precisely this approach, as a means of overcoming legal chaos and enhancing the internal structure of the public legal order in the context of judicial and law enforcement reforms, that facilitated rethinking of the organizational system for ensuring procedural aspects, specifically the institution of forensic examination. The use of philosophical-legal, managerial, and data-driven analytics has made it possible to propose radical changes, namely the reorganization of the current model through the institutional centralization of the activities of forensic scientists and the creation of a single central body to which all powers in the field of expert support for the justice system should be transferred.

The bulk of the analyzed documents covered the period from 2022 to 2026, which made it possible to assess the transformation of Ukraine's forensic science system amid martial law, European integration reforms, and the digitalization of public administration. The documentary basis of the study consisted of: 1) Ukrainian regulatory and legal acts (Laws of Ukraine, decrees of the President of Ukraine, resolutions of the Cabinet of Ministers of Ukraine) – 7 documents; 2) international regulatory and strategic documents and recommendations of international organizations (Council of the European Union, International Forensic Strategic Alliance, European Network of Forensic Science Institutes, ASCLD) – 10 documents; 3) scientific publications in the fields of public administration, forensic expertise, digital governance, quality management, and organizational development – 26 sources; 4) dissertations and monographs – 3 works; 5) official state registries and informational and analytical materials from the Ministry of Justice of Ukraine, the National Accreditation Agency of Ukraine, and the Office of the Prosecutor General – 10 sources; 6) official websites of Ukraine's forensic institutions containing information on international cooperation, personnel changes, and the implementation of digital technologies – 15 news items.

Particular attention is paid to the analysis of the following: the Law of Ukraine “On Forensic Examination” [12]; the Comprehensive Strategic Plan for Reforming Law Enforcement Agencies as Part of the Security and Defense Sector of Ukraine for 2023–2027 [13] and the Action Plan for its implementation [14]; documents related to the negotiation process for Ukraine's accession to the European Union [15]; [16]; [17]; [18]; [19]; the Register of Methods for Conducting Forensic Examinations of the Ministry of Justice of Ukraine [20]; the Register of Accredited Conformity Assessment Bodies the National Accreditation Agency of Ukraine [21]; the Unified State Register of Court Decisions of the State Judicial Administration of Ukraine [22]; and materials on expert support for the administration of justice and research activities of state specialized institutions.

The sources were selected based on the following criteria: a) direct relevance to the field of forensic science activities, public administration, or expert support for the administration of justice; b) relevance to the reform of Ukraine's forensic science system during the 2022–2026 period; c) the official nature of the source or its peer-reviewed status; d) the presence of information regarding organizational management, digitalization, accreditation, standardization, staffing, or international cooperation; e) the possibility of comparing Ukrainian experience with foreign practices.

## 4 Results

### 4.1 Current weaknesses in Ukrainian forensic governance

A current problem in the field of forensic science in Ukraine remains its historically formed fragmentation [23]. State specialized institutions are subordinate to various ministries and central executive bodies, in particular: the Ministry of Justice, the Ministry of Internal Affairs, the Ministry of Defense, the Ministry of Health, the Security Service, the State Border Guard Service [12]. The retention of a complex system of departmental regulations governing the conduct of forensic examinations runs counter to the principle of legal certainty, contributes to the proliferation of regulatory regimes, and also makes it impossible to ensure a single, predictable and consistently applied standard for forensic science activities [3]; [4]; [24]; [25]. Institutions subordinate to different central executive bodies apply different methodological approaches, which leads to inconsistent results in expert studies. In addition, this situation maintains the dependence of the expert environment on executive bodies, increases the risk of applying different criteria and levels of control [3]; [23]. This, in turn, weakens the guarantees of impartiality and objectivity of expert opinions, which are an important component of ensuring everyone's right to a fair trial.

The idea of separating forensic expertise from law enforcement, operational-search, counterintelligence activities, as well as from inquiry and pre-trial investigation within our country, is a long-standing one and it is high time to reform the extensive, multi-agency system of forensic expertise institutions into a single, stable and flexible organization of a new type [26]. It is proposed to form a single system of state forensic institutions, which would be responsible for conducting examinations, scientific and methodological work and training personnel in the field of forensic expertise and criminalistics [27]. At the same time, the units involved in the technical and forensic support of preliminary inquiries and pre-trial investigations (documenting traces, collecting evidence) should remain under departmental control, with the conduct of forensic examinations removed from their remit [27].

The emphasis is on the lack of centralized organizational and managerial support for forensic institutions, which creates numerous problems with the coordination of their activities [28]. Interdepartmental coordination in this area mostly takes place in a consultative format [2]; [29]. As a result, even if there are jointly agreed recommendations or approaches, their actual practical application may differ significantly depending on the departmental affiliation of the expert institution.

The absence of a single coordination center often leads to duplication of functions, contradictory decisions, and complicates the implementation of modern methods and international standards into national practice. The complex structure of this sphere, which includes multiple institutions with different areas of activity, different departmental subordination and a wide range of entities involved in expert provision of justice, necessitates the definition of a single body for the formation of public policy in the field of forensic science activity [19].

The implementation of the above is possible through the institutional separation of a single body to combine the functions of forecasting, planning, organization, coordination, and accounting of the field of forensic science activity. The creation of such a body would make it possible to centralize the management of forensic science institutions, introduce effective quality control of expertise, unify the procedures for certification and advanced training of forensic scientists, improve the regulatory and methodological support of the field, simplify interdepartmental interaction, create conditions for ensuring work in accordance with European standards, rationalize the use of expert potential, and increase trust in the expert conclusions in the judicial process. The process of state reform in the field of forensic science activities is administered by the Ministry of Justice of Ukraine.

Improving the quality of forensic expertise is a complex systemic problem, the solution of which requires efforts at the legislative, organizational, methodological, technological, informational, and personnel levels. Only a comprehensive approach through the modernization of the regulatory framework, active implementation of the latest scientific and technical achievements, constant improvement of the professional level of forensic scientists and integration into the European and international forensic community, are able to ensure a high level of reliability, authenticity and objectivity of forensic expert conclusions.

In general, the legislation of Ukraine does not establish a general obligation to apply international and European standards, harmonized in the form of the State Standard of Ukraine (DSTU), in the practice of state bodies and institutions [30]. Such implementation is voluntary, except in cases where authorized public authorities or administrations adopt special regulatory acts that establish the obligation to comply with the requirements of a specific standard.

The accreditation process of laboratories contributes to increasing their competence, maintaining their compliance with international requirements, and ensuring the trust of customers in the results of expert examination. The National Accreditation Agency of Ukraine (NAAU) accredited for compliance with the requirements of DSTU EN ISO/IEC 17025:2019 the following institutions: Ukrainian Scientific and Research Institute of Special Equipment and Forensic Expertise of the Security Service of Ukraine (ISEE SSU) until 20.05.2028; KSRIFE until 19.12.2028; NSC “Hon. Prof. M. S. Bokarius FSI” until 11.04.2029; Lviv Research Forensic Center of the Ministry of Internal Affairs of Ukraine until 19.01.2030; Kirovohrad Research Expert Forensic Center of the Ministry of Internal Affairs of Ukraine until 27.12.2030; Khmelnytsky Research Expert Forensic Center of the Ministry of Internal Affairs of Ukraine until 15.03.2028; Vinnytsia Research Expert Forensic Center of the Ministry of Internal Affairs of Ukraine until 22.12.2026; and others [21].

The experience of operation of the accredited laboratories gives grounds to conclude as follows:

- one of the priority tasks remains ensuring the flexibility and dynamism of the laboratory management system;
- it is necessary to constantly monitor the compliance of the management system with modern requirements of international standards and recommendations of the National Accreditation Agency of Ukraine (NAAU);
- for the development of the sphere of laboratory accreditation, it is important to conduct further research focusing on the implementation of modern requirements, which are in demand among customers;
- more attention should be paid to international standards regulating new research methods;
- it is advisable to actively apply risk management principles when planning laboratory activities and evaluating their results;
- it is recommended to organize regularly scientific and practical seminars directly in laboratories, dedicated to the development and implementation of modern research methods in the relevant field [31].

In conditions of martial law, as a special legal regime, forensic expertise plays a key role in documenting war crimes and gross violations of international humanitarian law. International standards of forensic science remain a fundamental benchmark for ensuring the quality and reliability of forensic expertise. However, their mechanical application without taking into account extraordinary circumstances is impossible. Adaptation of international standards should be based on preserving their basic principles while simultaneously introducing flexible implementation mechanisms through regulatory clarification of the procedures for forensic science activity in wartime.

An internationally recognized network of forensic science professionals – the American Society of Crime Laboratory Directors (ASCLD) [32], a member of the International Forensic Strategic Alliance (IFSA) [33] – identifies seven priorities for its work [32]; [34]; [35]. First of all, ethics and objectivity are highlighted, which exclude all obstacles to the independent ability to exercise professional judgment and impose on managers the responsibility to ensure that examinations are conducted in accordance with scientific principles and within the framework of legislative requirements. Emphasis is also placed on substantial and continuous funding of all needs to ensure the quality of expert opinions and save time on case work. The next priority is accreditation, in particular according to the ISO/IEC 17025 standard, which promotes quality. Education, training, certification, standardization and best practices are highlighted with an emphasis on scientific and methodological, technological support, financial efficiency, development of standard operating procedures and standardized use in all ASCLD member laboratories of unified research methods within one expert specialty. The sixth priority is supervision, as the advisory and supervisory infrastructure should consist of individuals well-versed in forensic science to address problematic issues and discredit unfounded criticism [32]; [34]. ASCLD strongly urges members to consider implementing ISO 21043, which is its seventh priority [32]; [35].

When studying the European legal framework for strategic planning related to the development of forensic science activities, attention should be paid to the EU Council's programmatic document –Action Plan for the European Forensic Science Area 2.0 (hereinafter referred to as the Action Plan 2.0) [36], the adoption of which is conditioned by the ENFSI Vision for the European Forensic Science Area by 2030 [37]. The first section is devoted to the development of technical and methodological capabilities of forensic science. The second section highlights the reliability of forensic findings and their role for the judiciary, strengthening cooperation between institutions and jurisdictions within and outside the EU, and focusing on the investigation of organised crime and terrorism. The third section pinpoints the study of the impact of human interaction on decision-making in forensic science activities, dissemination of the results of these studies, promotion of forensic scientists' professional competence, and strengthening the accreditation processes of laboratories.

The Comprehensive Strategic Plan for Reforming Law Enforcement Agencies as Part of the Security and Defense Sector of Ukraine for 2023-2027 (hereinafter referred to as the Comprehensive Strategic Plan) includes the formation of an independent and effective system of forensic expertise to ensure justice, the activities of law enforcement agencies and the prosecutor's office [13]. The above is planned to be achieved through a number of measures: creating a system of self-government of forensic scientists; introducing review of forensic expert opinions; determining deadlines for conducting forensic examinations; expanding access of individuals to forensic science services by equalizing the powers of private and public forensic scientists and increasing the level of competition between them; development of regulatory amd legislative acts to modernize the entire system of forensic expertise and determine the qualification requirements for forensic scientists; development of the system of forensic institutions with the establishment of appropriate safeguards to ensure the independence of the forensic service and the integrity of its staff; expansion of types of forensic expertise and improvement of their productivity; rejection of the outdated concept of “criminalistic expertise” [14].

The Roadmap on the Rule of Law (hereinafter referred to as the Roadmap) – a key document, the implementation of which is a mandatory condition for the start of official negotiations on Ukraine's accession to the European Union within Cluster 1 “Fundamentals of the EU Accession Process” under the negotiating Chapter 23 “Judiciary and Fundamental Rights” and Chapter 24 “Justice, Freedom and Security” [15]; [16]; [17]; [18] – sets out the strategic objective of improving law enforcement agencies' access to high-quality, impartial and prompt forensic expertise. To achieve this, the following measures are planned: unification of forensic science activities by establishing a single body responsible for formulating public policy; introduction of a single classification of forensic examinations and types of expert specialties; introduction of a single procedure for conducting examinations; expansion of the range of examinations to be conducted by private forensic scientists; specialization of forensic institutions to conduct research in certain areas, in particular in proceedings regarding corruption crimes; digitalization of forensic science activities; upgrading of the material and technical support of institutions for the work of highly qualified personnel; and accreditation in accordance with international ISO standards [19].

Thus, the two strategic documents of Ukraine contain somewhat different goals: the Decree of the President of Ukraine, dated 2023, in the Comprehensive Strategic Plan emphasizes the formation of an independent, effective system and the creation of self-government of forensic scientists; on the contrary, the Government Resolution, adopted in 2025, focuses in the Roadmap on high-quality, objective and rapid forensic expertise, and has no mention whatsoever of forensic scientists’ self-government.

### 4.2 Wartime operational pressures

The Unified Register of Pre-Trial Investigations has registered more than 230 thousand criminal proceedings related to war crimes [38]. Over 80% of investigations of complex serious crimes are conducted using forensic science expertise [39].

An analysis of 144 judgments passed between 23 May 2022 and 29 May 2026 under Article 438 of the Criminal Code of Ukraine indicates that a significant number of forensic examinations were conducted during the investigation of war crimes: forensic medical examinations – 146, psychiatric examinations – 29, molecular genetic examinations – 23, psychological examination – 11, weapon examination – 51, explosive and technical examination – 12, photographic and portrait examination – 16, commodity examination – 51, and others [22]. Forensic examinations were not ordered in only 29 cases [22].

Ukrainian forensic scientists work in extremely difficult conditions of a full-scale war. The most acute problem is a direct threat to life and health while working in areas where shelling continues or there is a danger of mining of territories. This requires the use of bulletproof vests, military or rescue unit escort, which significantly complicates the inspection of the scene, recording and analysis of evidence. Reducing the time that forensic scientists spend in dangerous areas is achieved due to modern technologies: unmanned aerial vehicles are used to quickly record damage; laser 3D scanning is used to create three-dimensional models, which allows for a remote basic analysis in laboratory conditions [40].

During situational studies of man-made disasters, particularly environmental assessments at the site of large-scale damage (for example, following the explosion at the Kakhovka Hydroelectric Power Plant, when the disaster zone extended across the Dnieper River from Kakhovka to the delta), forensic scientists often encounter significant uncertainty in the source data [41]. Under such conditions, a forensic experiment is conducted during the supplementary inspection. Additional data is obtained by surveying the most characteristic areas of the territory (flooded arboretums, livestock farms, oil spills, damaged sewer collectors, mineral fertilizer storage facilities, etc.) using unmanned aerial vehicles [41]. The survey area typically covers several dozen square kilometers. After processing photographic and video materials as well as instrumental measurements, the results are extrapolated to the entire damaged territory [41]. Although such an assessment has a certain margin of error, it is acceptable in wartime because it allows for a rapid determination of the approximate economic damage caused by the aggressor [41]. Even preliminary data from a situational environmental assessment is crucial for informing international organizations, securing political and humanitarian support, and justifying claims for reparations for environmental damage [41].

Logistical barriers caused by the destruction of transport infrastructure, road blockages, and the lack of safe routes lead to problems with the transportation of physical evidence and materials, which affects the quality and timeliness of forensic examinations [40]. A separate challenge is the preservation of examination objects, a significant part of which is damaged by artillery strikes, fires, explosions or other destructive factors. Forensic scientists often have to work with partially destroyed, incomplete, damaged materials, which complicates the elucidation of the truth and requires the use of modern digital technologies for data fixation and restoration – photogrammetry, 3D scanning, electronic databases. Additional difficulties are created by the forced relocation of forensic institutions. Due to active hostilities and destructions in certain regions, some state specialized institutions and their branches were forced to evacuate or temporarily cease work. This has led to concentration of a significant number of cases in safer regions, which caused an overload on forensic scientists, delays in conducting research, and violations of established procedural deadlines [40].

During wartime, the implementation of forensic science activities is significantly complicated due to the nature of the damage and the specifics of circumstances, which forces specialists to use non-standard methods and significantly adapt traditional approaches to the new realities. Structural and technical surveys turned out to be especially difficult. Unlike peacetime, when damage to structures is mostly associated with accidents or natural disasters, in wartime, destructions result from the impact of a shock wave, high temperatures during fires, seismic shocks from artillery shelling or air strikes. Therefore, forensic scientists have to rely on deep knowledge of building physics, the characteristics of military weapons and the behavior of building materials under excessive loads.

It is necessary to cooperate with the military experts in order to determine accurately the type and origin of the ammunition used and analyze traces of modern means of destruction – fragments of high-precision shells, rocket elements, kamikaze drones, and atypical fragments [40]. Of the 49 methodologies for examining traces, weapons and the circumstances of their use contained in the Register of Forensic Examination Methods, the use of 31 methods have been discontinued, 28 of them in 2022 [20]. As for the methodologies for examining explosives and explosion products, out of 26, the use of 23 methods was discontinued in 2022 [20].

The data in Table 1 give grounds to conclude that the arsenal of forensic examination methods is significantly outdated. The data in Table 2 confirm that the Ministry of Justice of Ukraine provides information on the total number of methods on its website [42], yet in fact, only 34% of the declared number are valid and allowed for use [20]. It is in the areas of forensic examination most in demand during wartime that this figure is the lowest, specifically: explosives and technical examination – 11%; trace evidence examination – 19%; weapons examination – 37%; examination of materials, substances and products – 9%; psychological examination – 23% and so on [20].

**Table 1:** Information on current methods used in forensic science examinations.

No.	Methodology name	Year of creation, amendments
*weapons examination*		
1	Methodology for identifying rifled short-barreled firearms (pistols and revolvers) by fired bullets	2009
2	Methodology for determining the ballistic coefficient of the striking elements of traumatic cartridges	2010
3	Methodology for identifying smooth-bore rifles by traces on shells	1973
4	Methodology for establishing the belonging of products to the category of hunting (sports) smooth-bore rifles	1999
5	Methodology for forensic examination of gas pistols and revolvers	2000
6	Methodology for forensic examination of shot pistols and revolvers	2002
7	Methodology for forensic examination of traumatic weapons with the firearm principle of projectile throwing (Methodology for forensic examination of self-defense pistols and revolvers (gas, shot, non-lethal action))	2012
8	Methodology for establishing the belonging of an object to a firearm and its suitability for shooting	2012
9	Methodology for comprehensive examination of firearms damage	2016
10	Methodology for establishing the belonging of an object to the ammunition of small arms and its suitability for firing	2012
11	Methodology for establishing the belonging of a bullet and a cylindrical sleeve to one cartridge by traces of their interaction with each other	2010
12	Methodology for determining the ballistic characteristics of low-velocity kinetic projectiles	2015
13	Methodology for identifying smooth-bore rifles by traces on projectiles (improved)	2023
14	Methodology for assessing the suitability of dynamic traces for identification studies (improved)	2023
15	Methodology for studying edged weapons and structurally similar products (improved)	2023
16	Methodology for forensic ballistic studies of the circumstances of a shot	2013
17	Methodology for forensic examination of mortars	2024
18	Methodology for conducting forensic examination in the process of investigating events related to the use of missile weapons	2025
*explosives and technical analysis*		
1	Study of shot products	1997
2	Methodology for studying the composition of explosives, explosion products using high-performance chromatography and mass spectrometry	2020
3	Methodology of forensic investigation of industrial and homemade electric detonators	2005

Compiled by the author based on [20].

**Table 2:** Data from the register of forensic examination methods.

No.	Type of examination	The total number of methods in the Register	Data on current methods used in forensic examinations	%
1	Handwriting and linguistic examination	92	33	36
2	Technical examination of documents	135	48	36
3	Weapon examination	49	18	37
4	Traceological examination	109	21	19
5	Explosive and technical examination	26	3	11
6	Photographic and portrait examination	15	4	27
7	Video and audio recording examination	36	18	50
8	Examination of materials, substances and products	424	37	9
9	Biological examination	42	14	33
10	Engineering and technical examination	212	147	69
11	Economic examination	111	63	57
12	Commodity examination	27	13	48
13	Examination in the field of intellectual property	20	19	95
14	Psychological examination	88	20	23
15	Artistic examination	18	7	39
16	Military examination	7	7	100
17	Examination of special technical means of secret information acquisition	5	5	100
18	Veterinary examination	5	5	100
19	Historical and archaeological examination	2	2	100
20	Gemological examination	1	1	100
21	Complex examination	27	17	63
	Total	1451	502	34

Compiled by the author based on [20].

Scientists insist on the need to periodically review the methodologies in order to identify outdated and duplicative ones [43]. If a methodology cannot be applied in expert practice due to changes in legislation, regulatory and technical acts, the scientific councils of state specialized institutions submit a proposal to the Coordination Council to discontinue the use of that methodology [44]. In case the Coordination Council decides to amend the methodology or to discontinue its use, the relevant data shall be entered into the Register by a structural unit of the Ministry of Justice [2]; [45]. Only in 2022, by decisions of the Coordination Council on Forensic Examination Issues under the Ministry of Justice of Ukraine dated January 28, 2022, and September 30, 2022, the use of 780 methodologies was discontinued [46]; [47].

This forces the forensic examination system to introduce new methods, modern technologies and adapted procedures. According to the Thematic plan of scientific and research works of scientific and research institutions of forensic examinations of the Ministry of Justice of Ukraine for 2026, 85 scientific research works are to be carried out by state specialized institutions of this Ministry in 2026. However, only 43 of them are expected to result in a methodology, i.e. 51% [48]. The rest are going to result in teaching and methodological guides or research and methodological manuals, monographs, and methodological recommendations [48]. Methodologies are being developed on the following topics: Development of a methodology for identifying rifled firearms using spent cartridges; Development of a methodology for conducting forensic molecular genetic studies using the ANDE 6C system; Development of a methodology for linguistic examination of offensive statements; Development of a methodology for forensic examination of facial images; Development of a methodology for identifying and analyzing combat toxic substances using instrumental methods; Development of a methodology for identifying and analyzing substances with radioactive properties using instrumental methods, etc. [48].

In order to improve existing and develop new methods of forensic examinations, determine the estimated value of construction objects and structures, study real estate objects, building materials, structures and relevant documents, receive scientific and methodological support when solving complex technical tasks, and train specialists, state specialized institutions conclude memorandums of cooperation and interaction with Kyiv National University of Construction and Architecture, the Public Union “Interstate Guild of Consulting Engineers”, etc. [49]; [50].

Preparation for various threat scenarios, as a prerequisite for ensuring the stability of the state, is implemented through creation of clear and detailed response plans, regular exercises and training events, as well as a targeted formation of a competent personnel reserve capable of ensuring the effective functioning of the system in crisis conditions [51]. The resilience of a system is characterized by its strength, reliability of internal elements, as well as vertical, horizontal and other types of connections that allow it to withstand both internal and external loads.

### 4.3 Digital transformation needs

Among the key problematic issues of forensic expertise in the field of blockchain technologies, the challenges caused by the war unleashed by the aggressor state against Ukraine have become particularly acute. To conduct aggressive actions in cyberspace, the enemy uses all the advantages of blockchain i.e. decentralized nature, high anonymity, transaction speed and ease of use. This is manifested, in particular, in the creation of “cold” wallets for collecting funds, launching phishing resources, organizing cryptocurrency pyramids, fraudulent smart contracts and other malicious blockchain schemes. To effectively counter such threats in the digital environment, specialized knowledge and skills are required, and it is forensic scientists who possess them [52].

The role of digital technologies for recording and storing data has significantly increased [53]. Modern tools allow information to be stored even when physical objects are completely or partially destroyed. Photogrammetry and 3D scanning are widely used to create accurate virtual models of destroyed buildings or event locations. Unmanned aerial vehicles are actively used to survey large areas, providing aerial photography from above. All collected data is systematized in secure electronic storage, which guarantees its preservation, accessibility for investigators and courts, and prevents future information loss [40].

The Ministry of Justice of Ukraine has implemented a pilot version of an artificial intelligence-based service on the platform of the Research Center for Independent Forensic Expertise, intended for persons ordering forensic examinations or expert studies. AI significantly simplifies the process of preparing documents – it helps to draw up an application or a resolution on the appointment of a relevant examination. This tool is designed for a variety of users: individuals, lawyers, investigators and other interested parties. Its main goal is to reduce the number of typical errors that often occur at the stage of document preparation. In particular, AI provides support in the following aspects: a correct choice of the type of examination that best meets the needs; accurate definition of objects to be examined; clear formulation of questions addressed to the forensic scientist [54].

Digitalization as a tool for resilience helps reduce dependence on physical infrastructure. Decentralized information systems, such as blockchain, can digitize routinized work processes, providing tighter control and increasing the certainty of behavior [9]. Currently, the pace of development of the digital environment is significantly ahead of the adaptation of forensic science methods, updating the regulatory framework, and technical equipment for conducting examinations. Such inconsistency creates risks of reducing the effectiveness of evidence and violating the rights and freedoms of participants in the proceedings. In this regard, the areas of integration of modern high technologies into forensic practice, increasing the level of digital competencies of forensic scientists, monitoring their workload, implementing an automated system for assigning expertise to increase the transparency of all forensic science activity, unifying approaches to processing digital evidence, forming a single digital environment for forensic science activity, and expanding international cooperation are of key importance [25]; [53].

When analyzing the European legal regulation of the digitalization of forensic support of justice, it is worth referring to the Action Plan 2.0, which defines the vision and strategic priorities for the development of forensic science in the European Union for the period until 2030 [36]. The first section, dedicated to new technologies, envisages a widespread use of artificial intelligence, digitalization of expert processes, active implementation of innovative technologies and automated processes at all stages (from the crime scene to the court). This directly relates to the harmonization of forensic standards in Europe, which is important for Ukraine in the context of European integration and cooperation with ENFSI.

The roadmap emphasizes the creation of a single digital space for carrying out forensic science activity, integrated with the Unified Judicial Information and Telecommunications System (UJITS) and other state registers, information from which is necessary for conducting examinations [19].

### 4.4 Personnel and leadership instability

Today, courts, law enforcement agencies and prosecutors’ offices are putting forward fundamentally new requirements for forensic expertise [3]. The tasks facing state specialized institutions in modern conditions require an active search for ways to increase the efficiency of the system, optimize its organization and legal support. This necessitates further improvement of public administration mechanisms in this area.

It is important to pay attention to the personnel policy of the Ministry of Justice of Ukraine implemented in recent years in subordinate state specialized institutions. Since 2023, four directors have been changed in the National Scientific Center “Hon. Prof. M. S. Bokarius Forensic Science Institute” [55]; [56]; [57]; [58]; [59], and five in Dnipro Scientific Research Institute of Forensic Expertise [59]; [60]; [61]; [62]; [63]. KSRIFE was headed by O. Ruvin from 2011, but in January 2025 a new director was appointed [64]. Accordingly, the structure of the institution, its deputies and heads of structural units are being changed, which causes a turnover of specialists.

Such high staff turnover indicates the presence of systemic managerial changes and, at the same time, raises the issue of staff stability in the field of forensic science activities. The websites of the Ministry of Justice of Ukraine and its subordinate specialized state institutions do not provide any explanation for the dismissals.

From the perspective of public administration, frequent leadership changes may caused by a combination of several factors: reforms to the forensic science system; efforts to modernize management mechanisms; increased oversight of institutional performance; and a drive to renew personnel in response to new management and security challenges. At the same time, excessive turnover at the leadership level can create risks of losing institutional continuity, disrupting the stability of internal organizational processes, and weakening the personnel capacity of institutions.

The issue of the psychological and emotional strain on the heads of state expert institutions warrants special attention. The death on July 4, 2023, of the deputy director and head of the Kyiv branch of the National Scientific Center “Hon. Prof. M. S. Bokarius Forensic Science Institute” of the Ministry of Justice of Ukraine, Doctor of Law, Professor, and Honored Lawyer of Ukraine V. L. Fedorenko directly at his workplace [59]. He was appointed to this position on December 27, 2022; prior to that, from July 12, 2016, to December 26, 2022, he served as director of the Research Center for Forensic Expertise on Intellectual Property Issues at the Ministry of Justice of Ukraine [59]. Speculation has circulated in the public sphere regarding the possible impact of prolonged psychological pressure and a conflict-ridden organizational environment on the official's condition [59].

The conditions of martial law, a significant increase in the number of forensic examinations, the need to document war crimes, limited resources, and constant personnel changes have placed an additional burden on the leadership of the forensic science system. In the absence of effective psychological support mechanisms and a stable organizational environment, this can negatively impact both the quality of management decisions and the overall state of the personnel system. For the work of forensic scientists, which relies heavily on specialized expertise, academic traditions, and the continuity of professional practice, such trends may pose risks of a decline in the quality of expert support for the administration of justice.

In addition, staff turnover negatively affects the strategic planning of expert institutions. Frequent changes in management teams complicate the implementation of long-term programs for modernization, digitalization, international integration, and the upgrading of infrastructure. Every new management team typically develops its own vision of development priorities, which can lead to fragmented reforms and a lack of consistent management policy. Current trends indicate the need to strike a balance between updating management approaches and maintaining the institutional stability of the forensic science system.

Scientists conclude that the powers of the head of a state specialized institution are regulated in various ways by departmental orders and instructions, and the status in the criminal procedural legislation of Ukraine is not defined. They propose to enshrine the head's rights, obligations, as well as prohibited actions, in a separate article of the Law of Ukraine “On Forensic Expertise” [65].

What is important for the field of forensic science activity, being a connecting link between science and practice, is not the use of employees’ physical strength, but their intellect, which belongs to the specialist, not the employer. The modern head of a forensic institution should be a leader of a new dimension with a broad horizon of knowledge and well-versed in contemporary models of strategic thinking. Such a person should demonstrate a creative approach to management, be an organizer and an active participant in scientific activity. It is mandatory for the manager to constantly improve his qualifications for effective management of a highly professional team that is constantly developing. It is the manager who implements a comprehensive approach to improving the quality of forensic expertise and minimizing expert errors within a particular institution. It depends on the manager how effectively the measures prescribed by legislation will be taken, as well as whether other tools for improving its quality will be used.

### 4.5 International integration and standards

Membership of state specialized institutions in ENFSI, accreditation according to ISO/IEC 17025 and ISO/IEC 17020, international scientific ties with forensic institutions, joint conferences and seminars, exchange of trainees and scientific information unite expert schools of different countries, contribute to increasing resilience through external resources and knowledge. The results of a survey conducted by V. Shepitko indicate almost unanimous support for the idea of international cooperation in forensic science activities: 95.3% of respondents (164 people) consider it necessary. The most promising areas of such interaction, according to the respondents, are: membership in reputable international associations, in particular ENFSI (54.7%, 94 experts); receiving software and technical assistance from foreign colleagues (48.3%, 83 people); conducting joint research (44.8%, 77 respondents); organizing joint scientific and practical events (40.7%, 70 experts); involving foreign specialists in commission expertise (36.6%, 63 people); improving the quality of forensic services through international experience (30.2%, 52 respondents); inviting foreign forensic scientists to directly perform expertise (27.3%, 47 people); and joint publication of scientific works (26.7%, 46 experts). Other forms of cooperation were mentioned by only 1.7% of respondents (3 people) [66].

Daily, forensic scientists conduct research related to war crimes. This has led to the accumulation of an unprecedented amount of practical experience, which has no analogues in countries not at war. This experience is already of significant value to the global forensic community, allowing foreign colleagues to expand their own methodological base, adapt international standards to emergency situations, and increase their readiness to work in high-risk areas. Dnipropetrovsk Scientific Research Institute of Forensic Expertise held a seminar for police officers from Lithuania, Latvia and Estonia entitled “A Comprehensive System for Recording Losses from Damaged and Destroyed Property as a Result of the Russian Federation's Military Aggression” [67].

Forensic science institutions cannot function effectively without close interaction with the relevant organizations, laboratories and centers of other countries. Thus, since 2023, Panacea Cooperative Research (Spain) and the National Scientific Center “Hon. Prof. M. S. Bokarius Forensic Science Institute” (hereinafter referred to as NSC “Hon. Prof. M. S. Bokarius FSI”) have been cooperating on the use of free Skeleton-ID software [68]. In 2024, Kyiv Scientific Research Institute of Forensic Expertise (KSRIFE) received a license to use the eyesCloud3D product free of charge, after signing a memorandum of cooperation with Ecapture Research and Development S.L. Photogrammetry will facilitate the documentation and analysis of evidence, in particular in crimes of the Russian Federation in Ukraine [69]. KSRIFE experts demonstrate to colleagues from the Japan International Cooperation Agency the study of alcoholic beverages and tobacco products as a tool for combating the shadow market, and undergo training at the International Anti-Corruption Training in Japan [70].

The importance of nuclear forensics has grown significantly, as Russian forces have repeatedly carried out missile and artillery strikes on areas located near nuclear power plants [71]. At the Idaho National Laboratory, Ukrainian specialists are trained by American experts having many years of experience in nuclear forensics [72].

Of particular importance for increasing the efficiency of forensic science expertise is cooperation within international organizations of forensic scientists, in particular the European Network of Forensic Expert Institutions, which contributes to solving the tasks of standardization and harmonization of forensic scientist practice, organization of thematic scientific and practical events, as well as exchange of methodological materials, technologies and reference information. The signing of the Memorandum of Understanding by the International Commission on Missing Persons with the NSC “Hon. Prof. M. S. Bokarius FSI” is aimed at searching for and identifying missing persons, providing evidence for criminal justice processes [73]. A special role is played by molecular genetic examinations, which are carried out to identify people killed in combat, in mass graves or as a result of large-scale destruction. Often, biological material is seriously damaged by fire, water or mechanical factors, which makes it necessary to use the most modern methods of molecular genetics to obtain reliable results.

The support of international partners in updating laboratory equipment is also important. Representatives of the Japan International Cooperation Agency were demonstrated the updated capabilities of KSRIFE: 3D modeling and digital visualization for the reconstruction of damaged objects and complex events; photo-portrait identification and accurate comparative studies; work with mobile phones and digital media with the recovery and extraction of critically important information. Japanese-made equipment is an integral part of expert solutions [70].

### 4.6 Recommended reforms

The current stage of social development is marked by continuous transformations, a high degree of uncertainty, significant risks and hidden potential opportunities. In such conditions, the task of finding innovative methods and resources aimed at strengthening the institutional stability and financial capacity of the forensic sphere is particularly acute.

1. First of all, it is necessary to carry out a deep transformation of the institutional model of administration of the field of forensic science activity, since a formal assignment of the status of the sole subject of public policy formation in this area to a certain executive body will not automatically provide new mechanisms of public administration. Ensuring the impartial development of rules in the field of forensic science activity and their uniform application to all participants in the forensic services market is enabled by the delimitation of key functions: public policy formation, regulation and control, direct management of expert institutions [11]. Through a clear distribution of powers, the risks of creating unequal conditions for different subjects of forensic science activity are minimized [11].

From the perspective of public administration, a centralized coordinating body should become a key element of the institutional framework for managing the field of forensic science activities. Its operations should be aimed at creating a unified organizational, methodological, and informational framework in the field of forensic examination, ensuring the coordination of activities between state and non-state entities engaged in expert activities, and enhancing the effectiveness of implementing public policy in the field of expert support for the administration of justice.

One possible means of implementing this model could be the establishment of a separate central executive body or a specialized state agency in the field of forensic expertise. The jurisdiction of such a body should include coordinating the activities of expert institutions, accrediting and certifying forensic scientists, maintaining state registries, developing and approving methodologies for conducting expert examinations, organizing scientific and methodological support, international cooperation, and the digital transformation of forensic science activities. Futhermore, a centralized body can ensure the monitoring of the quality of forensic examinations, the development of uniform standards for the professional training and continuing education of forensic scientists. An important tool for implementing the concept of centralized management is the development of a unified regulatory and legal framework for the operation of the entire forensic science system. One of the key areas in the coordinating activities of the centralized body should be ensuring the independence of forensic science from undue influence by law enforcement agencies and other entities with a vested interest in the results of expert examinations.

The expected impact of establishing a centralized coordinating body is to enhance the efficiency of the forensic science system, ensure consistency in methodological approaches, and improve the quality of forensic examinations. Centralized coordination functions will minimize the duplication of responsibilities among agencies, optimize the use of resources, and ensure a higher level of organizational cooperation among entities involved in forensic science activities. This will also create the conditions for the implementation of modern digital technologies, the development of scientific research, and the adaptation of the national forensic science system to international standards.

2. The standardization of forensic examination practices is a necessary prerequisite for the creation of a unified professional framework in the field of forensic science and entails the introduction of uniform requirements for the methodologies used in conducting forensic examinations, procedures for documenting investigation results, the professional training and certification of forensic scientists, and the internal quality control system within forensic institutions. The existence of accredited forensic institutions is a key prerequisite for the international recognition of forensic findings and for fostering mutual trust between expert bodies in different countries. Furthermore, accreditation creates the conditions for the introduction of modern technologies and innovative methodologies, as well as for improving the efficiency of the public administration of forensic science activities. At the same time, the process of standardizing and accrediting forensic institutions requires an appropriate regulatory framework, the establishment of effective quality control mechanisms, and the maintenance of a balance between the standardization of expert activities and the procedural independence of forensic scientists.

The tools for implementing the unification policy include harmonizing the national system of forensic science activities with international standards in the field of conformity assessment and quality management, as well as developing and approving uniform methodological guidelines and standards for conducting forensic examinations. This involves creating a centralized system for the state registration and verification of methodologies, establishing uniform approaches to the preparation of expert opinions, and defining criteria for evaluating the quality of forensic examinations. The unification of methodological support will help reduce discrepancies in conducting forensic examinations and ensure the stability of law enforcement activities.

A key element of the reform is the creation of an effective accreditation system for forensic institutions, regardless of their form of ownership. The accreditation should be based on transparent procedures for evaluating staffing, technical equipment, the level of professional training of forensic scientists, and the effectiveness of internal quality control systems. It is advisable to introduce regular monitoring of the activities of accredited institutions, external audit mechanisms, and periodic confirmation of their compliance with established standards.

The harmonization of standards and the implementation of an effective accreditation system are also crucial for ensuring the independence and professional accountability of forensic scientists. Uniform standards for professional practice create the conditions for an objective assessment of the quality of expert analyses, minimize subjective influence, and establish a consistent approach to forensic scientists’ professional ethics.

The expected impact of implementing a policy to unify standards and accredit forensic institutions is improved quality, reliability, and evidentiary value of expert opinions. The introduction of uniform standards will ensure a consistent level of forensic expertise across different institutions, help minimize errors, and enhance the efficiency of court proceedings.

3. Forensic science should function as an independent component of the justice system, rather than as a supporting tool for law enforcement. A forensic scientist should perform the procedural role of an independent investigator who provides specialized knowledge to establish the factual circumstances of a case, rather than acting as a component of the prosecution mechanism. This is why the institutional separation of the expert system from investigative bodies is viewed as one of the prerequisites for strengthening guarantees of a fair trial and ensuring the adversarial principle.

One way to implement this approach could be the creation of a unified, independent system of state forensic institutions with a centralized coordinating body that is not part of law enforcement or pre-trial investigation agencies. At the same time, the forensic units of law enforcement agencies may retain the functions of providing operational support for investigative actions, securing evidence, and collecting evidentiary information without conducting full-scale forensic examinations.

An important element of the reform should be the standardization of the legal framework governing the operations of all entities engaged in forensic science activities, regardless of their form of ownership or institutional affiliation. It is also expedient to establish professional self-governance within the forensic community and to ensure meaningful representation of independent and private forensic experts in advisory and coordinating bodies.

The expected impact of separating forensic activities from those of investigative bodies lies primarily in enhancing the independence and objectivity of forensic examinations, strengthening the credibility of expert opinions in court proceedings, and reducing the risk of procedural abuses.

4. The national digital environment for forensic science activities should comprise a set of interconnected information resources, electronic registers, automated systems and digital services designed to support the full cycle of organizing and conducting forensic examinations. The use of unified information platforms will ensure an appropriate level of quality control for expert investigations, simplify access to methodological materials, improve the level of analytical processing of information, and ensure effective monitoring of the activities of forensic institutions. The creation of a unified digital environment should be accompanied by the introduction of modern cyber-security mechanisms, user identification and authentication systems, as well as clear regulatory frameworks governing access to information resources in the field of forensic expertise. A key advantage of creating a unified digital environment is the standardization and harmonization of forensic procedures.

The tools for implementing this concept could include the creation of a centralized state digital platform for forensic science activities, integrated with e-justice systems, the information systems of pre-trial investigation agencies, and state registries. It would be advisable to include in the structure of such a platform an electronic registry of forensic examinations, an automated system for assigning expert studies, a unified registry of certified forensic scientists, a digital repository of examination methodologies, as well as integrated modules for working with multimedia evidence, 3D models, remote sensing results, and other digital materials.

An important element of the digital environment is the automation of processes for collecting and analyzing expert information. The use of artificial intelligence, big data an alytics, machine learning, and automated image processing systems makes it possible to improve investigation efficiency and optimize information retrieval. In the long term, this will enable the rapid identification of typical characteristics of objects under investigation, automated damage analysis, comparison of digital models, and the generation of supporting analytical conclusions for forensic scientists.

The legal framework for the digital transformation of forensic science activities is of critical importance to the functioning of a unified digital environment. It is essential to establish the legal status of electronic evidence and digital models and to regulate the use of automated information systems.

The expected impact of establishing a unified national digital environment consists in improving the efficiency of the forensic science system, reducing the time required to conduct expert examinations, and ensuring a higher level of reliability and objectivity of expert conclusions. The digitization of forensic activities creates the conditions for integrating the Ukrainian forensic science system into the international information and scientific-methodological space, which is an important prerequisite for adapting to European standards of justice.

5. The stabilization of leadership within forensic institutions should be based on the principles of professionalism, political neutrality, transparency in appointment and dismissal procedures, and institutional independence. A key factor in this process is the introduction of competitive selection mechanisms for senior management, the establishment of clear criteria for evaluating their performance, and the assurance of an appropriate level of accountability for the institution's management outcomes. Stability in the leadership helps to establish a consistent personnel policy, develop the scientific and methodological foundations of forensic science, ensure the continuity of international cooperation, and implement innovative approaches in the work of forensic institutions. The presence of professionally trained and stable leadership also has a positive impact on the level of trust in the forensic system on the part of the public, judicial bodies and participants in the judicial process.

The development of a professional talent pool for the system of forensic institutions is of paramount importance. The implementation of training and professional development programs for managerial staff, as well as the advancement of specialized management education in the field of forensic science activities, will contribute to the creation of a stable and professional management environment. It is also advisable to introduce modern approaches to strategic management, risk-based management, and digital administration into the operations of forensic institutions. It is essential to improve the system for separating managerial and procedural functions. The development of internal control and strategic planning systems plays a significant role in ensuring management stability. The creation of well-defined programs for the development of forensic institutions, the identification of key performance indicators, and the implementation of mechanisms for monitoring performance will ensure the consistency of management decisions and enhance organizational resilience.

Stable and professional leadership creates the conditions for implementing long-term development strategies, improving human resource policies, and enhancing the quality of expert research.

6. The effective functioning of the forensic science system today is impossible without active cooperation with international professional organizations, foreign forensic institutions and research centers, which facilitates the exchange of expertise, the adoption of modern methodologies and the harmonization of national standards with international requirements. Integration into the international forensic community requires the harmonization of national legislation, the standardization of methodological approaches, the improvement of accreditation procedures for forensic institutions, and ensuring that the work of forensic scientists complies with international quality standards. Expanding international cooperation requires appropriate organizational and legal frameworks, the development of institutional capacity within forensic institutions, and the training of specialists capable of working in accordance with international standards. An important aspect of this process is improving the language training of forensic scientists [4], ensuring access to international academic resources, and creating conditions for Ukrainian experts to participate in international professional programs and internships.

A key tool for developing international cooperation is the organization of joint educational and research programs, internships, international conferences, and training sessions for forensic scientists. Exchanging professional experience with foreign specialists helps enhance forensic scientists’ professional competence, adopt innovative research methods, and foster a modern culture of forensic practice.

International cooperation in the field of digital forensics and information exchange is of particular importance. The establishment of secure channels for exchanging forensic information, DNA test results, ballistics records, digital models, and other data creates the conditions for the effective investigation of transnational crimes. In the long term, this will contribute to the creation of an integrated international expert information environment capable of ensuring operational cooperation between forensic institutions in different countries.

International cooperation is particularly relevant in the context of documenting and investigating war crimes, crimes against humanity, and violations of international humanitarian law. The participation of international experts and specialized organizations in conducting comprehensive investigations helps ensure objectivity and a robust evidentiary basis for national and international judicial institutions. At the same time, such cooperation facilitates the adaptation of Ukraine's forensic science system to international criminal justice mechanisms.

International integration provides an environment for modernizing the material and technical infrastructure of forensic institutions, developing scientific potential and innovative technologies, and strengthening the institutional capacity of Ukraine's forensic science system.

Ukrainian forensic expertise attracts the attention of the international professional community due to its high level of professionalism and current scientific and practical developments. Positive experience of interaction with foreign colleagues allows us to predict that in the near future the domestic forensic system will move to a fundamentally new format of work – with intensive use of the latest technological solutions, modern approaches to research and full digitalization of expert procedures.

## 5 Conclusions

The experience of forensic institutions' operation under martial law has shown that the absence of a unified coordination mechanism hinders rapid responses to new challenges, whereas centralized strategic management enhances the system's resilience and the efficient use of resources. The focus is on the need to transition from fragmented management models to integrated institutional systems capable of ensuring the unity of public policy, standardization of procedures, and effective coordination among all actors.

The paper justifies the need to unify standards for forensic science activities and to establish independent accreditation mechanisms for expert institutions. Harmonizing procedures with international quality standards not only enhances the reliability of expert opinions but also lays the groundwork for fostering mutual trust between national expert systems and international judicial institutions.

The proposed mechanisms for ensuring the autonomy of the forensic sector and safeguarding its independence from undue influence by law enforcement agencies hold significant potential for adaptation in other legal systems. The institutional separation of forensic science activities from pre-trial investigation bodies helps strengthen the principles of objectivity, impartiality, and fair trial.

Leadership instability and the lack of a professional talent pool have a negative impact on the quality of management and institutional memory. Emphasis is placed on the need for competitive selection of managers, clear criteria for evaluating their performance, and the creation of management training programs.

Equally important is the experience of digital transformation in forensic science activities, which has become particularly relevant in the context of documenting the consequences of armed aggression, war crimes, and large-scale destruction of infrastructure. The introduction of a unified digital environment and the use of artificial intelligence, photogrammetry, remote sensing, 3D modeling, and electronic registries open up new opportunities for improving the efficiency and reliability of forensic investigations.

The practice of documenting war crimes, crimes against humanity, and large-scale environmental damage demonstrates the importance of fostering cooperation between expert institutions and international organizations. The participation of foreign forensic scientists in conducting research, developing methodologies, and applying international standards has helped ensure an adequate evidentiary basis for national and international legal proceedings.

The formulated recommendations extend beyond the national context and hold universal significance for the development of modern forensic science systems. Ukraine has in fact become a testing ground for new organizational, digital, and international legal mechanisms governing forensic science activities amid unprecedented security challenges. The results obtained can be used by other jurisdictions as a practical guide for building independent, technologically advanced, institutionally robust, and internationally integrated forensic justice-support systems capable of functioning effectively both under normal conditions and during periods of crisis-driven transformation.

## Ethics approval and consent to participate

The given manuscript is a review article. Therefore, there is no requirement for ethical approval or participation consent. This article does not contain any studies with human participants or animals performed by the authors.

## Consent for publication

The author gives full consent for the publication of the article on approval.

## Availability of data and material

All required data pertaining to the manuscript has been already provided. There is no supplemental data.

## Funding

This research received no specific grant from any funding agency in the public, commercial, or not-for-profit sectors. This research received no external funding. This study was not funded.

## Declaration of competing interest

The authors declare that they have no known competing financial interests or personal relationships that could have appeared to influence the work reported in this paper.
